# Cat and Dog Ownership in Early Life and Infant Development: A Prospective Birth Cohort Study of Japan Environment and Children’s Study

**DOI:** 10.3390/ijerph17010205

**Published:** 2019-12-27

**Authors:** Machiko Minatoya, Atsuko Araki, Chihiro Miyashita, Sachiko Itoh, Sumitaka Kobayashi, Keiko Yamazaki, Yu Ait Bamai, Yasuaki Saijyo, Yoshiya Ito, Reiko Kishi, The Japan Environment and Children’s Study Group

**Affiliations:** 1 Hokkaido University Center for Environmental and Health Sciences, Sapporo 060-0812, Japan; 2 Hokkaido University Faculty of Health Sciences, Sapporo 060-0812, Japan; 3 Department of Social Medicine, Asahikawa Medical University, Asahikawa 078-8510, Japan; 4 Faculty of Nursing, Japanese Red Cross Hokkaido College of Nursing, Kitami 090-0011, Japan

**Keywords:** pet ownership, child development, birth cohort, ASQ-3

## Abstract

Contact with companion animals has been suggested to have important roles in enhancing child development. However, studies focused on child development and pet ownership at a very early age are limited. The purpose of the current study was to investigate child development in relation to pet ownership at an early age in a nationwide prospective birth cohort study: the Japan Environment and Children’s Study. Associations between cat and dog ownership at six months and infant development at 12 months of age were examined in this study. Infant development was assessed using the Ages & Stages Questionnaires^TM^ (ASQ-3) at 12 months. Among participants of (Japan Environment and Children’s Study) JECS, those with available data of cat and dog ownership at six months and data for the ASQ-3 at 12 months were included (*n* = 78,868). Having dogs showed higher percentages of pass in all five domains measured by ASQ-3 (communication, gross motor, fine motor, problem-solving, and personal-social) compared to those who did not have dogs. Significantly decreased odds ratios (ORs) of developmental delays were observed in association with having dogs in all fix domains (communication: OR = 0.73, gross motor: OR = 0.86, fine motor: OR = 0.84, problem-solving: OR = 0.90, personal-social: OR = 0.83). This study suggested that early life dog ownership may reduce the risks of child developmental delays.

## 1. Introduction

Childhood is a crucial phase in its contribution to the quality of health, emotional well-being, learning, and behavior across the life span [1]. However, studies of child development have largely been limited to relationships and interactions with other humans. Studies of the human–animal interaction have proposed that there are health benefits associated with pet ownership. Pet ownership may provide emotional protection from the stresses and strains of life, and it may encourage a more active life. Contact with companion animals has been suggested to have important roles in enhancing child development [2,3]. The psychological benefits of attachment to pets have been found for a variety of pet animals, particularly cats [4] and dogs [5]. Several studies have found a link between pet ownership, pet attachment, positive attitudes to animals, compassion, empathy, and prosocial behavior [6,7,8,9,10,11]. For many children, companion animals are likely powerful motivators for learning [12] and development [12,13]. Although pet ownership may have the potential to positively influence child development, these relationships have received little attention, and a need for research in this area has been recognized [12,14]. Especially, studies focused on child development and pet ownership at a very early age and are limited. This could be partly due to the limited availability of developmental assessment tools at early ages. 

The Ages & Stages Questionnaires^TM^ (ASQ-3) is a developmental screening tool widely used by clinicians, researchers, and intervention programs around the world [15]. The ASQ-3 pinpoints developmental progress in children between the ages of 1 to 66 months. Because this tool is parent-completed, it provides an efficient and cost-effective method of collecting information regarding children’s development without the need for trained experts and considered to be an appropriate method for large prospective cohort study. 

The purpose of the current study was to investigate child development in relation to pet ownership at an early age. In this study, associations between cat and dog ownership at six months and infant development at 12 months of age were examined using the data from a nationwide prospective birth cohort study; the Japan Environment and Children’s Study (JECS). 

## 2. Materials and Methods

### 2.1. Study Design

The JECS is an ongoing nationwide prospective birth cohort study in Japan. JECS study is conducted at 15 regional centers (Hokkaido, Miyagi, Fukushima, Chiba, Kanagawa, Koshin, Toyama, Aichi, Kyoto, Osaka, Hyogo, Tottori, Kochi, Fukuoka, and Minami Kyusyu/Okinawa) in Japan. Details of the JECS project have been described elsewhere [16,17,18]. Briefly, pregnant women were recruited between January 2011 and March 2014. Eligibility criteria for participation included residing in the study area at the time of recruitment, an expected delivery date after August 2011, comprehension of the Japanese language, and completing the self-administered questionnaire. In total, 104,065 fetal records were included in the cohort, including multiple births. The present study used the dataset jecs-an-20180131, which was released in January 2018 and revised in December 2018.

### 2.2. Ethical Statement

The JECS protocol was approved by the Ministry of the Environment’s Institutional Review Board on Epidemiological Studies and by the Ethics Committees of each participating institution (Appendix A) (ethical project identification code: Kanken19-117). All participants provided informed, written consent in accordance with the Declaration of Helsinki.

### 2.3. Study Participants

**Figure 1:**
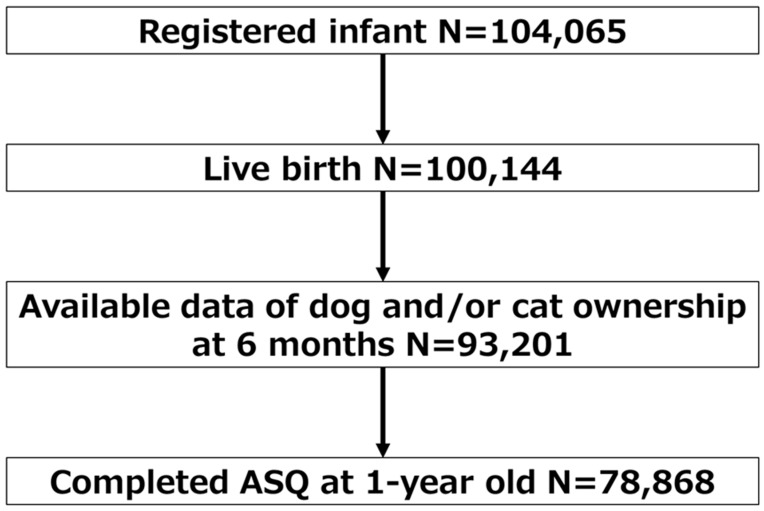
Selection of study population.

Of the 104,065 fetal records included in the cohort, 100,144 were live birth. Among participants of JECS, those with available data of dog and/or cat ownership at six months and data for the ASQ-3 at 12 months were included in this study (*n* = 78,868) (Figure 1).

### 2.4. Self-Administered Questionnaires

Details of the self-administered questionnaire in this study have been described previously [16,17]. Briefly, maternal smoking and drinking at the 2nd/3rd trimesters, maternal and paternal education, and annual household income during pregnancy were obtained from the M-T2 questionnaire (answered by pregnant women at the 2nd/3rd trimesters); parity was obtained from the Dr-T1 questionnaire (medical records transcripts at the 1st trimester); and maternal age at delivery, delivery mode, infant sex, gestational age, and birth weight were obtained from the Dr-0m questionnaire (medical records transcripts at delivery). Marital status was obtained from the C-6M questionnaire (answered by mothers at 6 months of postpartum). Duration of breastfeeding, and maternal mental illness (a Japanese version of the Kessler 6 (K6) scale) were obtained from the C-1Y questionnaire (answered by mothers at 12 months of postpartum). Information regarding pet ownership including cats and dogs was obtained from the C-6M questionnaire (answered by mothers at 6 months of postpartum). 

### 2.5. Outcome Definitions

The ASQ-3 is comprised of 21 age-specific questionnaires for children ages 1 to 66 months to assess children’s progress in five developmental domains (communication, gross motor, fine motor, problem-solving, personal-social). Each of the 5 domains has 6 questions, resulting in 30 items for each age-interval. Each item describes a skill, ability, or behavior to which the parent responds “yes” (10 points), “sometimes” (5), or “not yet” (0). Parents sometimes omit items when they are unsure of how to respond or because they have concerns about their child’s performance of the item. The ASQ-3 scores were not calculated if there were three or more omitted items in a given domain. In the case of one or two omitted items, an adjusted total domain score was calculated by adding the averaged item score either once for one omission or twice for two omissions. Children who may potentially be at-risk for developmental delays at each age-interval are identified by comparing their scores to cutoff scores. The J-ASQ-3 cutoff scores for each domain were; communication = 15.64, gross motor = 21.49, fine motor = 34.50, problem-solving = 27.32, personal-social = 21.73 based on the original ASQ-3 [19]. In this study, those who completed the J-ASQ-3 12-months questionnaire at 11 months 0 days through 12 months 30 days of ages were strictly included. According to recommend ASQ-3 procedures, adjusted age was used to determine the appropriate ASQ-3 for children who were preterm (<37 weeks of gestational age). 

### 2.6. Statistical Analyses

ASQ-3 scores of each domain were dichotomized based on the cutoff scores of each domain. Chi-squared test was used to determine whether there was a significant difference in the frequency of pass/fail of ASQ-3 scores at 12 months of age each domain in having or not having cats and dogs at 6 months of age. *t*-test was used to compare the mean value of maternal age, birth weight, and gestational age among those who had cats and dogs and those who did not. Logistic regression models were used to investigate infant developmental delays in association with having cats and dogs. The models were adjusted for maternal and paternal education, maternal smoking during pregnancy, annual household income during pregnancy, maternal mental illness, and duration of breastfeeding based on the previous literature and correlation between these variables and exposure and outcome. Since there were a small number of missing values for each variable, analyses included only those without missing variables. *p* < 0.05 was considered as statistically significant. Statistical analyses were performed using SPSS version 24 (IBM, Armonk, NY, USA).

## 3. Results

Table 1 shows the characteristics of parents and infants included in this study (*n* = 78,868) and comparison of characteristics between those who had cats and dogs. The pet ownership for cats and dogs at six months was 6358 (8.1%) and 11,934 (15.1%), respectively. Prevalence of maternal smoking at the 2nd trimester was higher in those who had cats and dogs. Both maternal and paternal education levels and annual household income were lower in those who had cats and dogs. Prevalence of possible maternal mental illness was higher in those who had cats but not dogs. Duration of breastfeeding was shorter in those who had cats and dogs. 

Table 2 shows the distribution of the ASQ-3 12-months questionnaire scores in association with having cats and dogs at six months of age. The mean scores of each domain were 37.68 for communication, 42.78 for gross motor, 48.21 for fine motor, 42.35 for problem-solving, and 37.03 for personal-social, respectively. Numbers of infants who failed each domain were 5633 (7.1%) for communication, 11,368 (14.3%) for gross motor, 8133 (10.2%) for fine motor, 12,505 (15.7%) for problem- solving, 13,755 (17.3%) for personal-social. Those who had cats showed higher percentages (83.7%) of pass in the personal-social domain compared to those who did not have cats (82.5%). Having dogs showed higher percentages of pass in all five domains measured by ASQ-3 compared to those who did not have dogs. 

Table 3 shows infant developmental delays at 12 months of age in association with having cats and dogs at six months of postpartum. There was no significant association between having a cat and infant developmental delays of any domains. Contrary, significantly decreased odds ratios (ORs) were observed in association with having a dog in all five domains. ORs and 95% confidence intervals (CIs) for each domain were 0.73 (0.67, 0.80) for communication, 0.86 (0.81, 0.92) for gross motor, 0.84 (0.78, 0.90) for fine motor, 0.90 (0.85, 0.96) for problem-solving, 0.83 (0.79, 0.88) for personal-social, respectively. Further analysis of three groups (only cat ownership, only dog ownership, and both cat and dog ownership) were conducted, and the results are shown in supplemental Table S3. 

## 4. Discussion

In this study, we found that having dogs at six months was associated with decreased risks of infant showing developmental delays at 12 months in five domains of the ASQ-3. However, the same was not observed among those who had cats. This was the first report on early childhood pet ownership and child development based on the large-scale prospective birth cohort study data.

The mean scores of the ASQ-3 in this study were relatively lower compared to the recent cohort study (*n* = 318) as well as US national data [20]. The mean scores were also lower compared to the original scores from the ASQ manual. As it was discussed in the recent literature from Japan, for the questionnaires for younger children, the scores tended to be lower for the J-ASQ-3 [21]. This is consistent with other developmental assessment tools that showed that Japanese children generally develop slower than American children until about two years of age [22]. 

The ownership of cats and dogs in this study was 8.0% and 15.0%, respectively, and this is comparable to the data from Japan Pet Food Association that reported the prevalence of cat and dog ownership in Japan was 9.78% and 12.64%, respectively, in 2018 [23]. In this study, socio-demographic characteristics of participants with cats or dogs were less educated parents and less household income, and maternal age at delivery was significantly younger, which was similar to previous studies [24,25]. In general, higher parental education and parental social class, and the younger the mother was at delivery, were related to less pet ownerships. This was only found in dog ownership but not in cat ownership. 

Our findings on pet ownership and child development are in line with findings from other studies. A systematic review found evidence for an association between pet ownership and a wide range of emotional health benefits, such as self-esteem and reduced depression from childhood pet ownership [26]. A recent study from Japan suggested that pet ownership in toddlerhood may contribute to the development of emotional expression of children [24]. The same study found that the prevalence of children with poor emotional expression were in the protective direction for owning dogs; contrary, the direction was opposite for cat ownership [24]. Infants could learn and develop skills of expression from interacting with their dogs and result in a lower risk of having communication and personal-social problems. Interaction with dogs may potentially help infants to have better gross and fine motor development. For example, a study of dog-assisted therapies and activities in the rehabilitation of children with cerebral palsy and physical and mental disabilities reported that the children improved their abilities to use their bodies according to their capabilities [27]. Another study showed that the presence of a therapy dog would affect the performance of a set of gross motor skill tasks for a mixed group of language-impaired and typical preschool children [28]. Children in these studies were older than those in the present study; however, these findings may be an indication of how beneficial interacting with dogs can be for the motor development of children. The previous studies suggested that early exposure to pets may contribute to child cognitive development [29] and may facilitate language acquisition and potentially enhance verbal skills in children [30]. The presence of animals has been shown to elicit immediate positive effects in testing situations of cognition, such as memory, categorization, and attention [28,31,32,33,34,35]. Problem-solving assessed in this study is related to cognitive function. Together with the previous studies, findings from this study suggested that the presence of dogs at home may contribute to child cognitive development even at a younger age. 

Literature included in the systematic review investigated various kind pets, including birds, fish, horses, and so on; however, the evidence for the impact of pets on child development was mostly found only with dogs [26]. It has been reported that children with pet dogs scored the highest on attachment [36]. Dogs are also more likely to read and adopt their behavior in response to human emotional signals [37]. These may explain why only ownership of dogs showed a significant decreased risk of children to have developmental problems. In addition, a study suggested that pet ownership may be associated with increased social skills, and the positive effect was only found in association with dog ownerships [38]. Research over the past 30 years [39] and a systematic review [40] indicated that dogs may offer physiological, emotional, social, physical support, and beneficial effect on a number of behavioral processes for children. 

Although the ownership of pets showed benefits, including better child development, certain negative consequences, such as zoonotic infections [41], allergy and asthma [42], bites and other injuries [43], and the psychological and emotional costs due to loss of pet [44] have been noted. In addition, children are at a greater risk of animal bites from a household pet [45,46], and children under five years of age are most at risk of serious injury [47,48]. 

The strengths of this study are that we used a dataset of large sample size, longitudinal design, and included a variety of different factors. Health influence in association with pet ownership has been discussed in case-control studies and cross-sectional studies [26,49]; however, these previous studies had limitations in study designs and sample size. In addition, some of the studies did not consider confounding factors. The current study was the first study to investigate child development in association with early life pet ownership in a prospective birth cohort study. The characteristics of parents and infants included in this study were compared to the whole cohort profile data and found to be similar to the original cohort population [16]. However, a comparison between the current study population (*n* = 78,868) and those who were excluded from the current study due to no data of pet ownership (*n* = 860) found some significant differences (Supplemental Tables S1 and S2). Percentage of maternal smoking was higher, and both maternal and paternal education levels and annual family income were lower among those who were excluded. These characteristics were also common among those who had cats and dogs (Table 1). This may indicate that the prevalence of pet ownership was possibly higher among those who were excluded. The mean ASQ-3 scores were higher among those who were excluded in all domains, which may indicate that less developmentally delayed children were in this group. These differences in characteristics may impact the findings from this study; however, the number of those excluded was small (1.1%); thus, the impact is also small even if it existed.

Some limitations of this study should be noted. First, the child developmental delays were assessed by a parental report. Even though the ASQ-3 is one of the most widely used screening tools for early childhood development, misclassification may have occurred. We have controlled for possible maternal mental illness based on the validated Japanese version of the K6 score [50] at 12 months postpartum as ASQ-3 scores may have been influenced by maternal mental problems. Second, the current study only investigated cat and dog ownerships but no other pets. Additionally, it is important to recognize that pet attachment may be more important in exerting potential health effects than pet ownership [51]. In this study, we could only examine the association between pet ownership and child development. For further studies, assessment of pet attachment should be considered. In addition to self-reported questionnaires, observational and behavioral methods would ensure the accuracy of findings. Third, other important confounding factors, such as the quality of children’s home environments, needed to be considered because children’s home environment has been linked with both the concurrent and longitudinal cognitive development of children [30,52]. The current study did not control for these confounding factors, which may possibly mask the true association between dog ownership and infant development. 

## 5. Conclusions

This study suggested that having dogs in early life may possibly reduce the risks of children to have developmental delays. Further longitudinal follow-up studies are necessary to elucidate associations between early life pet ownership and child development. 

## Figures and Tables

**Table 1:** Characteristics of participants.

		Mean ± SD or *n* (%)						
		All	Having Cat(s)			Having Dog(s)		
Parents		*n* = 78,868	Yes (*n* = 6358)	No (*n* = 72,510)	*p*-Value	Yes (*n* = 11,934)	No (*n* = 66,934)	*p*-Value
Maternal age at delivery (years)		30.7 ± 5.1	30.8 ± 5.1	30.7 ± 5.1	0.832	30.6 ± 5.0	30.8 ± 5.1	0.034
Parity	nulliparous	31,078 (39.4)	2503 (39.4)	28,575 (39.4)	0.788	4721 (39.6)	26,357 (39.4)	0.754
	multipara	32,986 (41.8)	3720 (58.5)	42,162 (58.1)		6932 (58.1)	38,950 (58.2)	
	missing	1908 (2.4)	135 (2.1)	1773 (2.4)		281 (2.4)	1627 (2.4)	
Maternal smoking at 2nd trimester	yes	2874 (3.6)	366 (5.7)	2508 (3.5)	<0.001	641 (5.4)	2233 (3.3)	<0.001
	no	75,051 (95.2)	5897 (92.7)	69,154 (95.4)	<0.001	11,128 (93.2)	63,923 (95.5)	<0.001
	missing	943 (1.2)	95 (1.5)	848 (1.2)		165 (1.4)	778 (1.2)	
Maternal drinking at 2nd trimester	yes	2134 (2.7)	191 (3.0)	1943 (2.7)	0.123	336 (2.8)	1798 (2.7)	0.415
	no	75,789 (96.1)	6086 (95.7)	69,703 (96.1)		11,447 (95.9)	64,342 (96.1)	
	missing	945 (1.2)	81 (1.3)	864 (1.2)		151 (1.3)	794 (1.2)	
Maternal education (years)	<13	26,859 (34.1)	2781 (43.7)	24,078 (33.2)	<0.001	4905 (41.1)	21,954 (32.8)	<0.001
	≥13	51,249 (65.0)	3506 (55.1)	47,743 (65.8)		6889 (57.7)	44,360 (66.3)	
	missing	760 (1.0)	71 (1.1)	689 (1.0)		140 (1.2)	620 (0.9)	
Paternal education (years)	<13	33,115 (42.0)	3220 (50.6)	29,895 (41.2)	<0.001	5940 (49.8)	27,175 (40.6)	<0.001
	≥13	44,570 (56.5)	3003 (47.2)	41,567 (57.3)		5750 (48.2)	38,820 (58.0)	
	missing	1183 (1.5)	135 (2.1)	1048 (1.4)		244 (2.0)	939 (1.4)	
Annual household income at 2nd trimester	<4	28,699 (36.4)	2581 (40.6)	26,118 (36.0)	<0.001	4440 (37.2)	24,259 (36.2)	<0.001
	≥4	44,361 (56.2)	3115 (49.0)	41,246 (56.9)		6205 (52.0)	38,156 (57.0)	
	missing	5808 (7.4)	662 (10.4)	5146 (7.1)		1289 (10.8)	4519 (6.8)	
Marital status	married	74,783 (94.8)	5952 (93.6)	68,831 (94.9)	<0.001	11,167 (93.6)	63,616 (95.0)	<0.001
	divorced/bereavement	529 (0.1)	72 (1.1)	457 (0.6)		149 (1.2)	380 (0.6)	
	missing	3556 (4.5)	334 (5.3)	3222 (4.4)		618 (5.2)	2938 (4.4)	
Maternal possible mental illness	yes (K6 ≥ 13)	2036 (2.6)	234 (3.4)	1802 (2.5)	<0.001	328 (2.7)	1708 (2.6)	0.205
	no (K6 < 13)	76,156 (96.6)	6067 (95.4)	70,089 (96.7)		11,493 (96.3)	64,663 (96.6)	
	missing	676 (0.8)	57 (0.9)	619 (0.9)		113 (0.9)	563 (0.8)	
**Infant**								
Sex	male	40,274 (51.1)	3255 (51.2)	37,019 (51.1)	0.721	6095 (51.1)	34,179 (51.1)	0.994
	female	38,572 (48.9)	3100 (48.8)	35,472 (48.9)		5835 (48.9)	32,737 (48.9)	
	missing	22 (0.0)	3 (0.0)	19 (0.0)		4 (0.0)	18 (0.0)	
Birth weight (g)		3041 ± 384	3037 ± 383	3041 ± 384	0.428	3041 ± 386	3041 ± 383	0.926
Gestational age (weeks)		39.4 ± 1.2	39.4 ± 1.2	39.4 ± 1.3	0.003	39.4 ± 1.2	39.4 ± 1.3	0.001
Delivery mode	vaginal	64,007 (81.2)	5157 (81.1)	58,850 (81.2)	0.796	9575 (80.2)	54,432 (81.3)	0.003
	cesarean	14,665 (18.6)	1191 (18.7)	13,474 (18.6)		2335 (19.6)	12,330 (18.4)	
	missing	196 (0.2)	10 (0.2)	186 (0.3)		24 (0.2)	172 (0.3)	
Duration of breastfeeding (months)	0	1978 (2.5)	205 (3.2)	1773 (2.4)	<0.001	346 (2.9)	1632 (2.4)	<0.001
	1–6	16,582 (21.0)	1646 (25.9)	14,936 (20.6)		3000 (25.1)	13,582 (20.3)	
	7–12	60,308 (76.5)	4507 (70.9)	55,801 (77.0)		8588 (72.0)	51,720 (77.3)	

Missing data: *n* (%), maternal age: *n* = 93 (0.1), birth weight: *n* = 41 (0.05). Chi-square test or *t*-test.

**Table 2:** Distribution of the ASQ-3 12-months questionnaire scores in association with having a cat and dog at six months of age.

			All	Having Cat(s)			Having Dog(s)		
ASQ Subscale	Cutoff			Yes	No	*p*-Value	Yes	No	*p*-Value
Communication	15.64	Mean ± SD	37.68 ± 13.43						
		Pass	73,216 (92.8)	5897 (92.8)	67,319 (92.9)	0.745	11,300 (94.7)	61,916 (92.6)	<0.001
		Fail	5601 (7.1)	458 (7.2)	5143 (7.1)		630 (5.3)	4971 (7.4)	
		Missing	51 (0.0)						
Gross motor	21.49	Mean ± SD	42.78 ± 17.55						
		Pass	67,556 (85.7)	5486 (86.3)	62,070 (85.6)	0.138	10,414 (87.3)	57,142 (85.4)	<0.001
		Fail	11,271 (14.3)	869 (13.7)	10,402 (14.4)		1511 (12.7)	9760 (14.6)	
		Missing	41 (0.0)						
Fine motor	34.50	Mean ± SD	48.21 ± 11.63						
		Pass	70,729 (89.7)	5729 (90.2)	65,000 (89.7)	0.236	10,840 (90.9)	59,889 (89.6)	<0.001
		Fail	8056 (10.2)	622 (9.8)	7434 (10.3)		1079 (9.1)	6977 (10.4)	
		Missing	83 (0.1)						
Problem-solving	27.32	Mean ± SD	42.35 ± 13.61						
		Pass	66,317 (84.1)	5346 (84.3)	60,971 (84.2)	0.965	10,158 (85.3)	56,159 (84.1)	0.001
		Fail	12,398 (15.7)	998 (15.7)	11,400 (15.8)		1754 (14.7)	10,644 (15.9)	
		Missing	153 (0.2)						
Personal-social	21.73	Mean ± SD	37.03 ± 14.57						
		Pass	64,927 (82.3)	5303 (83.7)	59,624 (82.5)	0.018	10,107 (84.9)	54,820 (82.2)	<0.001
		Fail	13,651 (17.3)	1032 (16.3)	12,619 (17.5)		1791 (15.1)	11,860 (17.8)	
		Missing	290 (0.4)						

*n* (%). Chi-square test.

**Table 3:** Infant development delays at 12 months of age in association with cat and dog ownership at 6 months of age.

	OR (95% CI)	
	Cat Ownership	Dog Ownership
Communication	0.94 (0.85, 1.04)	0.73 (0.67, 0.80) **
Gross motor	1.06 (0.98, 1.15)	0.86 (0.81, 0.92) **
Fine motor	1.07 (0.98, 1.18)	0.84 (0.78, 0.90) **
Problem solving	1.02 (0.95, 1.10)	0.90 (0.85, 0.96) *
Personal-social	1.07 (0.98, 1.16) +	0.83 (0.79, 0.88) **

Adjusted for maternal and paternal education, maternal smoke during pregnancy, annual household income during pregnancy, maternal mental illness (K6), duration of breastfeeding. + *p* < 0.10, * *p* < 0.05, ** *p* < 0.001.

## Supplementary Materials

The following are available online at https://www.mdpi.com/1660-4601/17/1/205/s1, Table S1: Comparison between the current study population and those who excluded from the current study due to no data of pet ownership. Table S2: Comparison of ASQ-3 scores between the current study population and those who excluded from the current study due to no data on pet ownership. Table S3: Infant development delays at 12 months of age in association with cat and dog ownership at 6 months of age.

## Appendix A

Members of the Japan Environment and Children’s Study (JECS) Group 2019; Michihiro Kamijima (principal investigator, Nagoya City University, Nagoya, Japan), Shin Yamazaki (National Institute for Environmental Studies, Tsukuba, Japan), Yukihiro Ohya (National Center for Child Health and Development, Tokyo, Japan), Reiko Kishi (Hokkaido University, Sapporo, Japan), Nobuo Yaegashi (Tohoku University, Sendai, Japan), Koichi Hashimoto (Fukushima Medical University, Fukushima, Japan), Chisato Mori (Chiba University, Chiba, Japan), Shuichi Ito (Yokohama City University, Yokohama, Japan), Zentaro Yamagata (University of Yamanashi, Chuo, Japan), Hidekuni Inadera (University of Toyama, Toyama, Japan), Takeo Nakayama (Kyoto University, Kyoto, Japan), Hiroyasu Iso (Osaka University, Suita, Japan), Masayuki Shima (Hyogo College of Medicine, Nishinomiya, Japan), Youichi Kurozawa (Tottori University, Yonago, Japan), Narufumi Suganuma (Kochi University, Nankoku, Japan), Koichi Kusuhara (University of Occupational and Environmental Health, Kitakyushu, Japan), and Takahiko Katoh (Kumamoto University, Kumamoto, Japan).
