# Supplementary material for: Cat and Dog Ownership in Early Life and Infant Development: A Prospective Birth Cohort Study of Japan Environment and Children’s Study
Source: Int J Environ Res Public Health. 2019 Dec 27;17(1):205. doi: 10.3390/ijerph17010205 (PMC6981655; doi:10.3390/ijerph17010205)
Supplement: Supplementary file 1 [file ijerph-17-00205-s001.pdf]

Supplemental Table 1 Comparison between the current study population and those who excluded from the current study due to no data of pet ownership

		Mean ± SD or n (%)		p-value
**Parents**		Current study (n=78,868)	Excluded (n=860)	
Maternal age at delivery (years)		30.7 ± 5.1	30.8 ± 5.2	0.645
Parity	nulliparous	31,078 (39.4)	336 (39.1)	0.844
	multipara	32,986 (41.8)	503 (74.0)	
	missing	1,908 (2.4)	21 (2.4)	
Maternal smoking at 2nd trimester	yes	2,874 (3.6)	93 (10.8)	< 0.001
	no	75,051 (95.2)	711 (82.7)	
	missing	943 (1.2)	56 (6.5)	
Maternal drinking at 2nd trimester	yes	2,134 (2.7)	29 (3.4)	
	no	75,789 (96.1)	778 (90.5)	0.139
	missing	945 (1.2)	53 (6.2)	
Maternal education	< 13	26,859 (34.1)	413 (48.0)	< 0.001
	≧ 13	51,249 (65.0)	400 (45.5)	
	missing	760 (1.0)	47 (5.5)	
Paternal education	< 13	33,115 (42.0)	467 (54.3)	< 0.001
	≧ 13	44,570 (56.5)	342 (39.8)	
	missing	1,183 (1.5)	51 (5.9)	
Annual household income at 2nd trimester	< 4	28,699 (36.4)	386 (44.9)	< 0.001
	≧ 4	44,361 (56.2)	351 (40.8)	
	missing	5,808 (7.4)	123 (14.3)	
**Infant**				
Sex	male	40,274 (51.1)	442 (51.4)	0.854
	female	38,572 (48.9)	418 (48.6)	
	missing	22 (0.0)	0 (0.0)	
Birth weight (g)		3041 ± 384	3021 ± 426	0.169
Gestational age (weeks)		39.4 ± 1.2	39.2 ± 1.5	< 0.001
Delivery mode	vaginal	64,007 (81.2)	680 (79.1)	0.133
	cesarean	14,665 (18.6)	177 (20.6)	
	missing	196 (0.2)	3 (0.3)	
Duration of breast feeding (months)	0	1,978 (2.5)	50 (5.8)	< 0.001
	1-6	16,582 (21.0)	304 (35.3)	

	7-12	60,308 (76.5)	506 (58.8)

Chi-square test or t-test.

Supplemental Table 2 Comparison of ASQ-3 scores between the current study population and those who excluded from the current study due to no data of pet ownership

			Mean ± SD		p-value
ASQ subscale	Cutoff		Current study (n=78,868)	Excluded (n=860)	
Communication	15.64	Mean ± SD	37.68 ± 13.43	40.9 ± 13.2	< 0.001
Gross motor	21.49	Mean ± SD	42.78 ± 17.55	44.5 ± 16.9	0.004
Fine motor	34.50	Mean ± SD	48.21 ± 11.63	48.9 ± 11.6	0.091
Problem-solving	27.32	Mean ± SD	42.35 ± 13.61	44.3 ± 13.5	< 0.001
Personal-social	21.73	Mean ± SD	37.03 ± 14.57	39.9 ± 14.4	< 0.001

t-test.

Supplemental Table 3 Infant development delays at 12 months of age in association with cat and dog ownership at 6 months of age

	OR (95% CI)		
	Only cat ownership	Only dog ownership	Both cat and dog ownership
Communication	1.14 (1.02, 1.28)*	0.75 (0.68, 0.83)**	0.65 (0.51, 0.83)*
Gross motor	0.93 (0.85, 1.02)	0.85 (0.79, 0.91)**	0.91 (0.78, 1.06)
Fine motor	0.93 (0.84, 1.03)	0.83 (0.77, 0.90)**	0.86 (0.72, 1.03)+
Problem solving	1.02 (0.94, 1.11)	0.92 (0.86, 0.98)*	0.83 (0.71, 0.96)*
Personal-social	0.96 (0.88, 1.04)+	0.84 (0.79, 0.89)**	0.79 (0.69, 0.92)*

Adjusted for maternal and paternal education, maternal smoke during pregnancy, annual household income during pregnancy, maternal mental illness (K6), duration of breast feeding.

+ $p < 0.10$, * $p < 0.05$, ** $p < 0.001$
